# HPV42 – ein als Niedrigrisiko‐Typ klassifiziertes humanes Papillomvirus mit onkogenem Potenzial

**DOI:** 10.1111/ddg.15981_g

**Published:** 2026-10-05

**Authors:** Sven Niklas Burmann, Ulrike Wieland, Steffi Silling, Frank Oellig, David Chromy, Julia Hyun, Thilo Gambichler, Alexander Kreuter

**Affiliations:** ^1^ Klinik für Dermatologie Venerologie und Allergologie Helios St. Elisabeth Klinik Oberhausen Universität Witten/Herdecke Oberhausen Deutschland; ^2^ Institut für Virologie Nationales Referenzzentrum für Papillom‐ und Polyomaviren Universität zu Köln Medizinische Fakultät und Uniklinik Köln Köln Deutschland; ^3^ Institut für Pathologie Mülheim an der Ruhr Deutschland; ^4^ Universitätsklinik für Dermatologie Medizinische Universität Wien Wien Österreich; ^5^ Klinik für Dermatologie Venerologie und Allergologie Helios St. Johannes Klinik Duisburg; ^6^ Klinik für Dermatologie Christliches Klinikum Unna Unna Deutschland; ^7^ Klinik für Dermatologie Klinikum Dortmund gGmbH Universität Witten/Herdecke Dortmund Deutschland

**Keywords:** Anogenitalwarzen, Condylomata acuminata, digitales papilläres Adenokarzinom, humanes Papillomvirus, HPV42, Plattenepithelkarzinom, Anogenital warts, condylomata acuminata, digital papillary adenocarcinoma, human papillomavirus, HPV42, squamous cell carcinoma

## Abstract

Das humane Papillomvirus (HPV) Typ 42 ist ein seltener, derzeit als *low‐risk* klassifizierter HPV‐Typ, der zum Papillomvirus‐Genus alpha gehört. In benignen vulvären Papillomen nachgewiesen, verursacht HPV42 überwiegend benigne Läsionen wie anogenitale Warzen. In sehr seltenen Fällen wurde HPV42 auch in invasiven genitalen Tumoren (insbesondere Zervixkarzinomen) detektiert. In der hier vorgestellten Fallserie konnte HPV42 in anogenitalen Warzen, einem digitalen Plattenepithelkarzinom in situ sowie in einem lymphogen metastasierten digitalen papillären Adenokarzinom (DPA) nachgewiesen werden. Bei letzterer Tumorentität wurde HPV42 kürzlich als auslösendes Agens entdeckt. HPV42 kann je nach infizierter Zellpopulation benigne (anogenitale Warzen und die seltene zervikovaginale *seborrhoic keratosis‐like lesion*) oder maligne (anogenitale und digitale Karzinome sowie das hochaggressive DPA) Läsionen auslösen. HPV42 sollte somit als potenziell karzinogen betrachtet werden. Mit dem aktuell verfügbaren nonavalenten HPV‐Impfstoff können mehr als 90% aller HPV‐induzierten Tumoren und anogenitalen Warzen verhindert werden, nicht jedoch HPV42‐induzierte Läsionen. Diese Tumoren werden somit durch die aktuellen HPV‐Impfprogramme nicht zurückgedrängt werden, auch nicht in Ländern mit hohen Impfraten.

## EINLEITUNG

Infektionen mit humanen Papillomviren (HPV) gehören zu den häufigsten Infektionen des Menschen. Aktuell sind mehr als 220 HPV‐Typen komplett klassifiziert worden und in verschiedene Genera (alpha‐, beta‐, gamma‐, mu‐ und nu) eingeteilt.^1^, ^2^ Die zum Genus alpha gehörenden circa 40 HPV‐Typen infizieren in erster Linie den Anogenitalbereich und können entsprechend ihrem onkogenen Potenzial in Hochrisiko/*high‐risk* (HR) und Niedrigrisiko/*low‐risk* (LR) HPV‐Typen unterteilt werden. HR‐HPV (zum Beispiel HPV16 und HPV18) verursachen anogenitale Karzinome und deren Vorstufen sowie Oropharynx‐Karzinome, wohingegen LR‐HPV (zum Beispiel HPV6, HPV11) benigne Läsionen wie Anogenitalwarzen (AGW) induzieren. In seltenen Fällen können LR‐HPV, wie bei Buschke‐Löwenstein‐Tumoren, auch intraepitheliale Neoplasien und invasive Karzinome verursachen.^3^, ^4^

HPV42 gehört zum Papillomvirus‐Genus alpha und hat eine enge phylogenetische Verwandtschaft mit HPV32, dem Auslöser der fokalen epithelialen Hyperplasie. HPV42 wird derzeit als LR‐HPV‐Typ eingestuft und wurde erstmalig 1982 in benignen vulvären Papillomen nachgewiesen.^5^ In größeren Untersuchungen konnte HPV42 in 1% – 2% der untersuchten AGW entdeckt werden.^6^, ^7^ In einer globalen Studie zu über 13 000 HPV‐induzierten anogenitalen Karzinomen konnte HPV42 nur in vier invasiven Tumoren nachgewiesen werden.^8^ Kürzlich wurde HPV42 in Tumorzellen von digitalen papillären Adenokarzinomen (DPA), einem sehr seltenen aggressiven Tumor mit überwiegender Lokalisation an Fingern und Zehen, identifiziert.^9^ Wir berichten über klinisch und histopathologisch unterschiedliche Läsionen bei drei Patienten, in denen HPV42 als Monoinfektion nachgewiesen werden konnte.

## FALLSERIE

### Patient 1

Ein 29‐jähriger Mann mit HIV‐Infektion, der Sex mit Männern hat, stellte sich aufgrund rezidivierender perianaler AGW in unserer Klinik vor. Die HIV‐Infektion (Stadium A2 nach CDC/WHO) war im Jahr 2018 diagnostiziert worden. Seitdem erfolgte eine antiretrovirale Therapie (ART) bestehend aus Raltegravir und Emtricitabin/Tenofovir‐Alafenamid. Zum Zeitpunkt der Erstvorstellung in unserer Klinik lag die CD4‐T‐Zellzahl bei 800/µl, und HIV‐RNA war im Plasma nicht nachweisbar (< 40 Kopien/ml). Klinisch zeigten sich multiple, bis zu 1 cm durchmessende AGW. Gemäß geltender AWMF‐Leitlinie wurde ein Screening auf anale Dysplasien angeboten und schließlich eine hochauflösende Anoskopie (HRA) durchgeführt, in der sich intraanal keine weiteren AGW oder Dysplasien zeigten.^10^ Histopathologisch imponierte in den perianal flachexzidierten Läsionen ein akanthotisch verbreitertes Plattenepithel mit leichter Basalzellhyperplasie sowie dichte Entzündungszellinfiltrate. In der immunhistochemischen Färbung für Ki‐67 imponierten einzelne suprabasale Zellkerne, die Färbung auf p16^INK4a^ fiel vollständig negativ aus (Abbildung 1a–d). In der anschließend aus einer Läsion durchgeführten HPV‐Typisierung konnte mittels alpha‐HPV‐gruppenspezifischer A6/A8‐PCR, die 43 HPV‐Typen abdeckt, eine Monoinfektion mit HPV42 nachgewiesen werden.^11^ Der Patient kommt weiterhin einmal jährlich zur HRA und ist seit drei Jahren rezidivfrei.

**ABBILDUNG 1:**
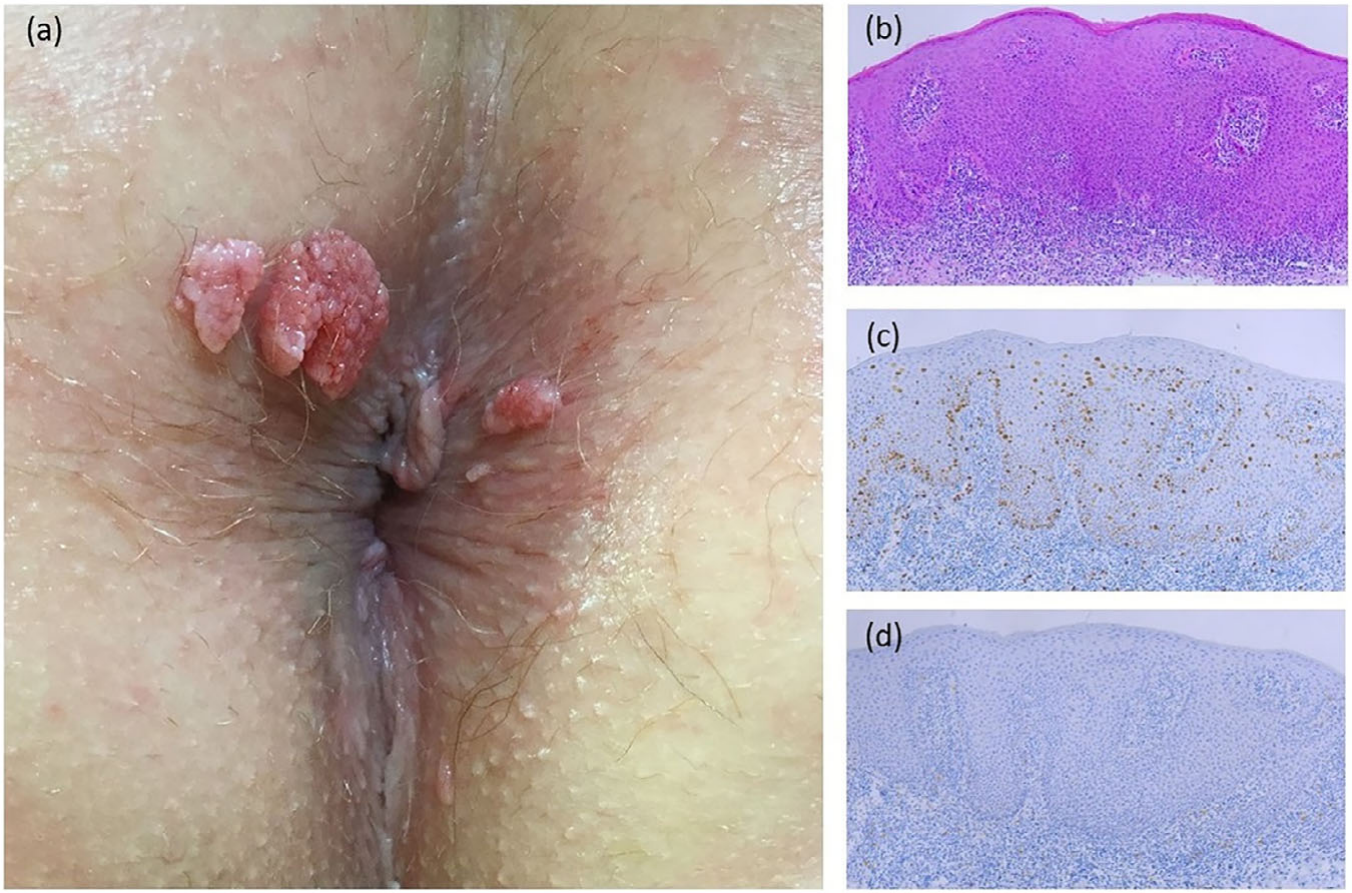
29‐jähriger Patient mit HIV und HPV42‐positiven Condylomata acuminata. (a) Bei der Erstvorstellung perianale Condylomata acuminata. (b) Histopathologie: akanthotisch verbreitertes, dysplasiefreies Plattenepithel mit leichter Basalzellhyperplasie und mäßig dichten Entzündungszellinfiltraten (Hämatoxylin‐Eosin‐Färbung, Originalvergrößerung × 100). (c) Immunhistochemisch hebt die Ki‐67‐Färbung vereinzelte suprabasale Zellkerne hervor (Ki‐67, × 100). (d) Die p16^INK4a^‐Expression ist im gesamten Epithel negativ (p16^INK4a^, × 100).

### Patient 2

Ein 65‐jähriger männlicher Patient stellte sich mit einer langsam wachsenden, seit circa 2 Jahren bestehenden schwärzlich pigmentierten, scharf abgegrenzten, verrukösen Plaque am Digitus IV der rechten Hand vor. Es lagen bis auf einen Nikotinabusus keine relevanten Begleiterkrankungen vor. Risikofaktoren wie erhöhte Lebenszeitdosis von ultravioletter Strahlung, Immunsuppression oder die Einnahme von photosensibilisierenden Substanzen wie zum Beispiel Hydrochlorothiazid wurden von dem Patienten verneint. Die histopathologische Aufarbeitung der chirurgisch entfernten Läsion ergab eine deutliche Verbreiterung der Epidermis mit atypischen Keratinozyten und Dyskeratosen bei fehlender Infiltration der Dermis. In der immunhistochemischen Färbung auf Ki‐67 imponieren positiv gefärbte Zellkerne bis in die oberen Zelllagen der Epidermis sowie eine bandförmige Expression von p16^INK4a^. In der Zusammenschau der Befunde wurde die Diagnose eines verrukösen Plattenepithelkarzinoms (SCC) in situ gestellt (Abbildung 2a–d). Nach histographisch kontrollierter kompletter Exzision konnte in der HPV‐Analyse mittels A6/A8‐PCR^11^ der Nachweis von HPV42 als Monoinfektion erbracht werden. In der letzten klinischen Nachsorge‐Untersuchung zwei Jahre nach Primärexzision des digitalen SCC in situ ist der Patient weiterhin tumorfrei.

**ABBILDUNG 2:**
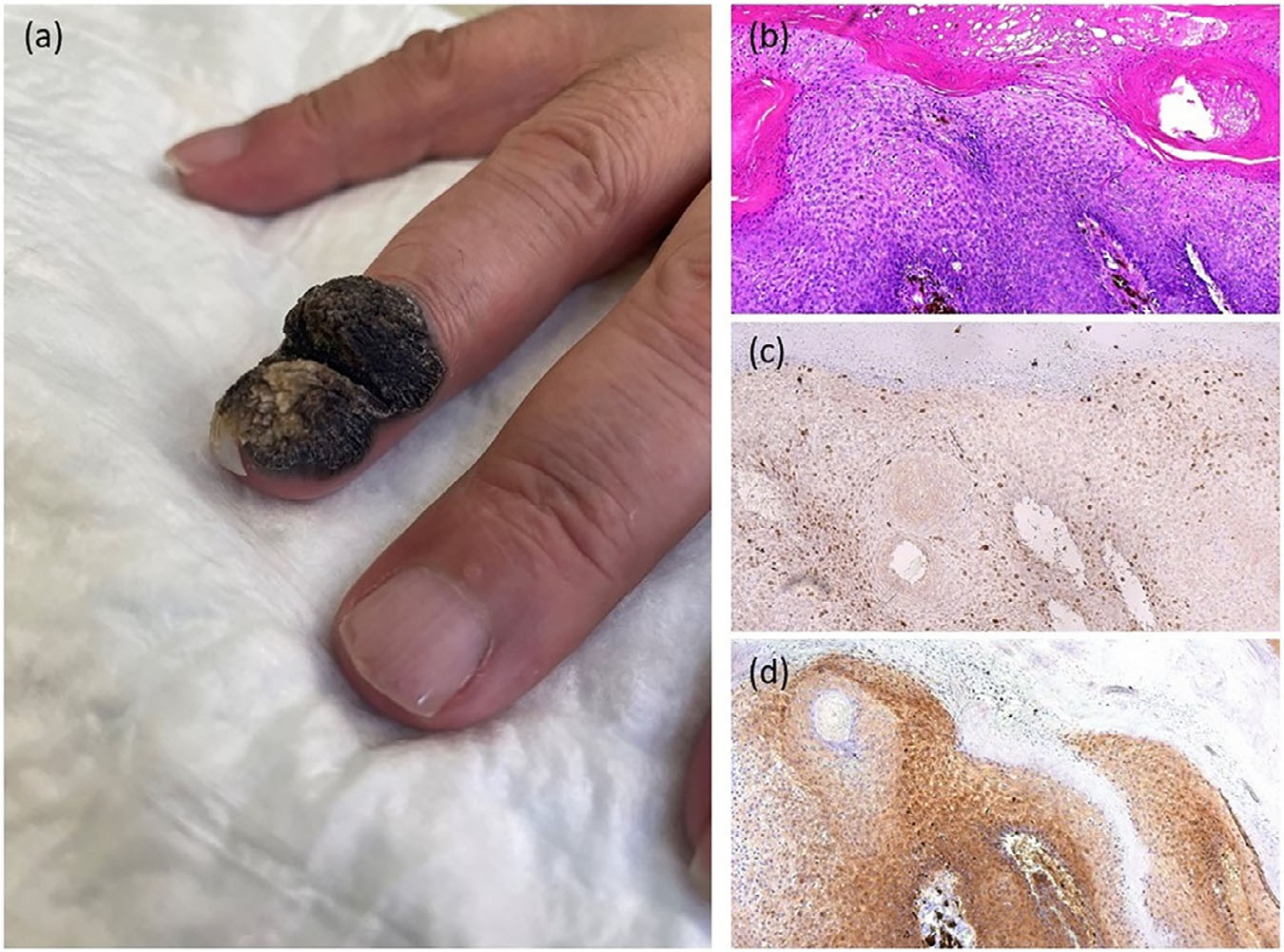
65‐jähriger Patient mit HPV42‐positivem peri‐ und subungualem Plattenepithelkarzinom in situ des Digitus IV der rechten Hand. (a) Klinischer Befund bei Erstvorstellung. (b) Histopathologie: Verbreiterung der Epidermis mit atypischen Keratinozyten und Dyskeratosen im gesamten Epithel ohne Dermisinfiltration (Hämatoxylin‐Eosin‐Färbung, Originalvergrößerung × 100). (c) Ki‐67‐Immunfärbung mit nukleärer Positivität bis in obere Epidermisschichten (Ki‐67, × 100). (d) Bandförmige (*block*) Expression von p16^INK4a^ (p16^INK4a^, × 100).

### Patient 3

Ein 52‐jähriger männlicher Patient stellte sich mit einer immer wieder blutenden, seit mehreren Monaten größenprogredienten Plaque am linken Mittelfingerendglied vor. Eine B‐Symptomatik bestand zum Zeitpunkt der Erstvorstellung nicht. Es lagen keine weiteren relevanten Vorerkrankungen wie zum Beispiel Immunsuppression vor. Palpatorisch fiel ein vergrößerter Lymphknoten der linken Axilla auf. Sonographisch zeigte sich ein hochgradig metastasenverdächtiger, vergrößerter Lymphknoten. In der apparativen Diagnostik mittels Computertomographie von Abdomen und Thorax sowie MRT des Schädels konnten keine weiteren pathologischen Befunde gefunden werden. Im Tumorexzidat des Fingers zeigten sich glandulär organisierte Tumorzellen mit überwiegend basophilen Zellpopulationen und fibrotischem Stroma. In der immunhistochemischen Färbung auf Ki‐67 imponiert eine deutlich erhöhte Proliferationsrate von mehr als 80% der Tumorzellen sowie eine durchgängige nukleäre und zytoplasmatische Expression in der p16^INK4a^‐Färbung (Abbildung 3a–e). Sowohl im Primärtumor als auch in der Metastase konnte mittels A6/A8‐PCR^11^ eine Monoinfektion mit HPV42 nachgewiesen werden. In der Zusammenschau der Befunde wurde die Diagnose eines lymphogen metastasierten digitalen papillären Adenokarzinoms gestellt. Nach Vorstellung im interdisziplinären Tumorboard wurde eine adjuvante Bestrahlung der linken Axilla mit einer Gesamtdosis von 45 Gy durchgeführt. Der Patient ist seitdem im 4. Folgejahr tumorfrei.

**ABBILDUNG 3:**
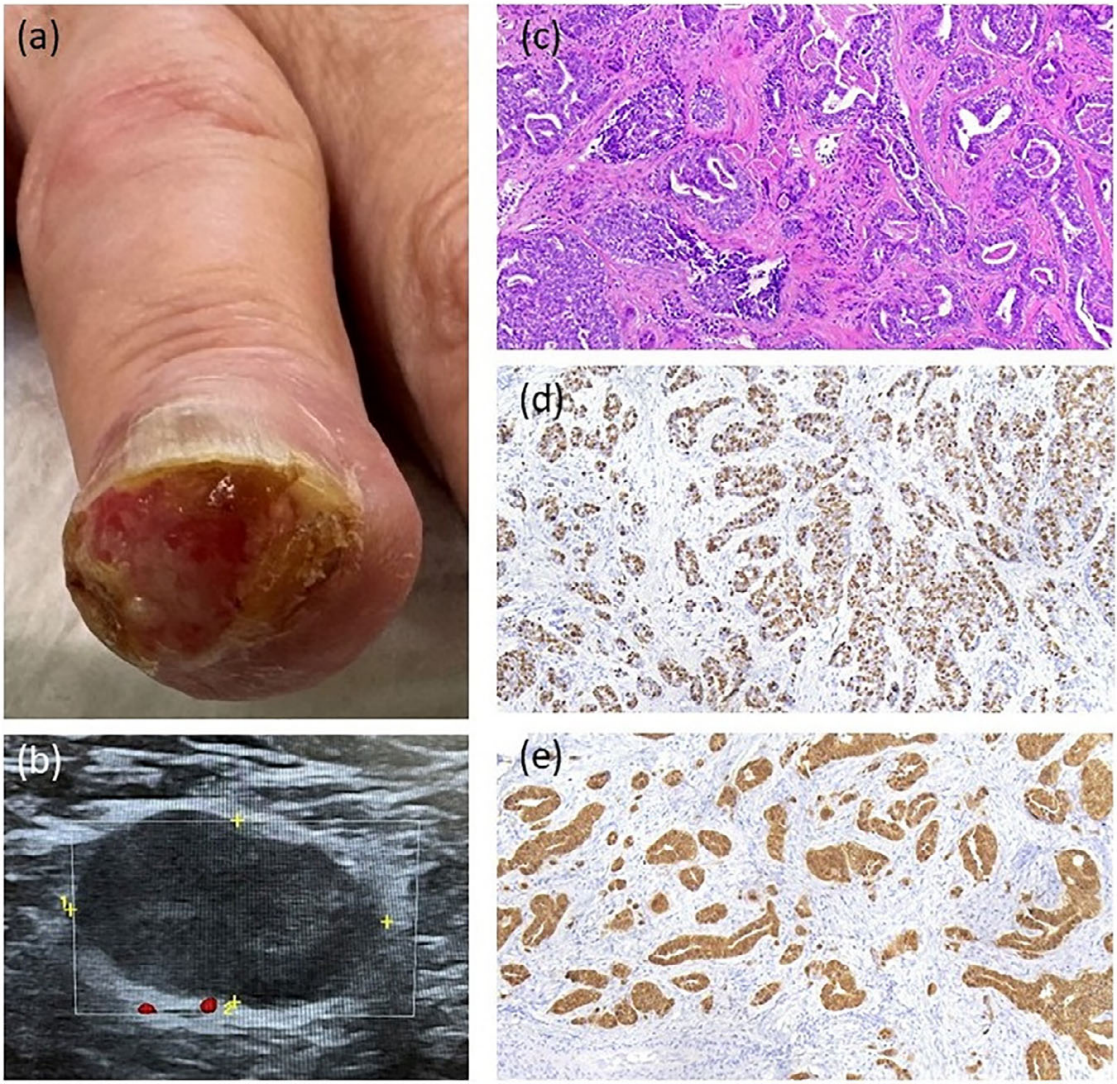
52‐jähriger Patient mit HPV42‐positivem digitalem papillären Adenokarzinom am Endglied des linken Mittelfingers. (a) Klinisch zeigt sich eine exophytisch wachsende verruköse Plaque. (b) Lymphknotenmetastase: In der hochauflösenden B‐Bild‐Sonografie der linken Axilla zeigt sich ein pathologisch veränderter Lymphknoten mit abgerundeter Morphologie und Längs‐/Querachsenverhältnis ≤ 2; die Binnenstruktur ist echoarm und inhomogen. Farbkodierter Doppler zeigt periphere Gefäßsignale ohne zentrale Hilusdurchblutung. Histopathologisch wurde die Lymphknotenmetastase bestätigt (nicht abgebildet). (c) Histopathologie des Primärtumors: glandulär organisierte Tumorzellen mit überwiegend basophilen Zellpopulationen und fibrotischem Stroma (Hämatoxylin‐Eosin‐Färbung, Originalvergrößerung × 100). (d) Ki‐67‐Immunfärbung mit deutlich erhöhter Proliferationsrate (> 80 % der Tumorzellen) (Ki‐67, × 100). (e) p16^INK4a^‐Färbung mit durchgängiger nukleärer und zytoplasmatischer Expression der Tumorzellen (p16^INK4a^, × 100).

## DISKUSSON

Die vorliegende Fallserie verdeutlicht, dass Infektionen mit dem (bisher noch) als LR‐HPV‐Typ eingestuften HPV42 neben benignen AGW auch Plattenepithelkarzinome beziehungsweise deren Vorstufen an den Fingern sowie das DPA, einen sehr seltenen aggressiven Tumor, auslösen können. HPV42 ist insgesamt ein seltener HPV‐Typ, der weltweit in Proben von Frauen mit unauffälliger Zytologie nur in 0,6% gefunden wurde. Dabei unterliegt die Prävalenz von HPV42 bei gesunden Frauen deutlichen geographischen Schwankungen, mit der höchsten Prävalenz von 1,2% in Afrika und nur 0,3% in Asien (HPV Information Centre; https://hpvcentre.net/datastatistics.php). In AGW konnte HPV42 in 1% –2% der Fälle nachgewiesen werden.^6^, ^7^ In dem hier präsentierten Fall eines Mannes mit HIV‐Infektion (Patient 1) konnte HPV42 als Monoinfektion in perianalen AGW detektiert werden. Seit langem ist bekannt, dass LR‐HPV in seltenen Fällen auch anogenitale Karzinome auslösen können. In einer Studie, in der Zervixkarzinome aus neun Ländern eingeschlossen wurden, konnte ein auslösender LR‐HPV‐Typ in weniger als 1% der analysierten 1739 Tumoren gefunden werden, wobei HPV42 nicht detektiert wurde.^1^ In einer anderen Studie mit 13 328 HPV‐induzierten Karzinomen wurde HPV42 nur in vier Fällen (3 Zervixkarzinome und 1 Analkarzinom) detektiert.^8^ Unsere Arbeitsgruppe konnte zeigen, das HPV42 bei einer immunkompetenten Patientin innerhalb kurzer Zeit in einem anatomischen Areal sowohl benigne AGW als auch hochgradige anale Dysplasien auslösen konnte.^12^ In der Literatur wurde eine Assoziation von HPV42 mit einer seltenen ungewöhnlichen zervikovaginalen Läsion, der *seborrheic keratosis‐like lesion* (SKLL), beschrieben.^13^, ^14^, ^15^, ^16^ Die SKLL unterscheidet sich morphologisch von anderen intraepithelialen Läsionen durch Ähnlichkeit mit kutanen seborrhoischen Keratosen (Akanthose und blande, teils basaloide Zellen mit squamoiden Abschnitten [squamous eddies] und interdigitierender Hyperpapillomatose) und fehlenden HPV‐induzierten zytopathischen Charakteristika wie zum Beispiel koilozytoider Zellmorphologie.^16^ In der bis dato größten Fallserie zur HPV‐Analyse bei SKLL konnte HPV42 in 13 von 17 (76,4%) Fällen nachgewiesen werden, wobei in zehn Fällen eine HPV42‐Monoinfektion und in drei Fällen eine HPV‐Koinfektion (HPV6, HPV16 und HPV61) vorlag. In zwei der untersuchten SKLL lagen zudem hochgradige Dysplasien mit typischem *block staining* in der p16^INK4a^‐Färbung vor.^16^

Im Gegensatz zu UV‐induzierten epithelialen Tumoren an Handrücken und Fingerstreckseiten werden sub‐ und periunguale SCC und SCC in situ der Finger häufig durch alpha‐HR‐HPV ausgelöst.^17^ Im hier beschriebenen Fall (Patient 2) wurde HPV42 in einem digitalen SCC in situ nachgewiesen; immunhistochemisch zeigte sich eine blockartige p16^INK4a^‐Expression (*block staining*), die einen kausalen Zusammenhang nahelegt. Unseres Wissens ist dies das erste durch HPV42‐induzierte digitale SCC in situ, das in der Literatur beschrieben wird. In früheren Untersuchungen unserer Arbeitsgruppe an 25 SCC und SCC in situ der Finger sowie der bisher größten Literatursuche mit über 100 HPV‐induzierten ungualen/periungualen SCC und SCC in situ (Mehrzahl der Fälle durch HPV16 induziert) konnte HPV42 in keinem Fall nachgewiesen werden.^17^, ^18^

Das DPA ist ein hochaggressiver, von den Schweißdrüsen ausgehender Adnextumor, der fast ausschließlich an Fingern und Zehen lokalisiert ist.^19^ Die Inzidenz des DPA wird mit 0,08/100 000 angegeben, wobei überwiegend Männer ab dem 50. Lebensjahr betroffen sind. Lokalrezidive nach mikrographisch kontrollierter Exzision sind häufig, ebenso lymphogene und pulmonale Metastasierung.^20^ Der von uns präsentierte Fall (Patient 3) entwickelte trotz mikrographischer R0‐Resektion des DPA eine Lymphknotenmetastase, ist jedoch nach Lymphknotenexstirpation und anschließender Radiatio der betroffenen Axilla seit mehr als 4 Jahren rezidivfrei. Der Nachweis von HPV42 mittels In‐situ‐Hybridisierung in primären und metastasierten DPA (alle 8 untersuchten DPA waren HPV42‐positiv und das positive Hybridisierungssignal war ausschließlich in den Tumorzellen nachweisbar) erfolgte erstmalig 2022 und konnte später in einem größeren Kollektiv bestätigt werden (45 von 47 [96%] der untersuchten DPA waren HPV42‐positiv).^9^, ^21^ Dabei konnten auch die Expression der HPV‐Onkogene E6 und E7 in Tumorzellen des DPA nachgewiesen werden. Auch bei fünf extrem seltenen, überwiegend genital lokalisierten, nicht‐akralen DPA wurde HPV42 nachgewiesen (5/5; 100%).^22^ Bayraktar et al.^23^ analysierten 14 Fälle von DPA mittels gezielter DNA‐/RNA‐Sequenzierung und Genexpressionsprofilierung und verglichen diese mit zehn Fällen von Hidradenomen. Zwei DPA wiesen Fusionsgene (CRTC3::MAML2 und TRPS1::PLAG1) auf, ohne ein einheitliches Mutationsmuster, zeigten jedoch eine erhöhte Expression von Genen im Zusammenhang mit Matrixumbau, Stoffwechsel und DNA‐Reparatur sowie eine Aktivierung des E2F‐Signalwegs. Alle getesteten DPA waren HPV42‐positiv, und die Immunhistochemie bestätigte eine erhöhte Expression von CD56, CDC20, SOX10 sowie eine Infiltration durch CD20‐positive B‐Zellen.^23^ Der Nachweis von HPV42 kann darüber hinaus hilfreich sein, das DPA von anderen morphologisch ähnlichen benignen Schweißdrüsentumoren wie dem Hidradenom oder dem tubulären Adenom abzugrenzen.^21^, ^24^, ^25^

Zusammenfassend kann HPV42 je nach infizierter Zellpopulation benigne (zum Beispiel AGW und die seltene zervikovaginale SKLL) oder maligne (anogenitale und digitale Karzinome sowie das hochaggressive DPA) Läsionen auslösen. Das bisher als LR‐HPV‐Typ klassifizierte HPV42 kann somit als (potenziell) onkogenes Virus beschrieben werden.^25^ Das E7‐Onkoprotein von HPV42 kann, ähnlich wie die E7‐Proteine der etablierten Hochrisiko‐HPV‐Typen HPV16 und HPV18, mit dem zellulären Tumorsuppressor‐Protein pRB interagieren, wie man auch an der starken p16‐Färbung (prä)maligner HPV42‐induzierter Tumoren erkennt.^8^, ^9^ Ebenfalls ähnlich wie bei HPV16 bindet das E6‐Onkoprotein von HPV42 an die E6AP Ubiquitin‐Ligase, dies führt aber bei HPV42 – im Gegensatz zu HPV16/18 – nicht zur Degradation des Tumorsuppressor‐Proteins p53.^8^, ^9^ Bei der Entstehung HPV42‐induzierter DPA spielt hingegen die Degradation des Tumor‐Suppressors PTPN14 durch HPV42‐E7 eine Rolle. Damit HPV42 sein onkogenes Potenzial entfalten kann, scheint auch die Art der infizierten Zellen relevant zu sein. Digitale papilläre Adenokarzinome gehen, im Gegensatz zu anogenitalen HR‐HPV‐induzierten Karzinomen, nicht aus Keratinozyten hervor, sondern aus ekkrinen Schweißdrüsenzellen, die an Händen und Füßen häufig vorkommen.^9^ Es ist bis dato noch unklar, ob und welche weiteren Faktoren (zum Beispiel Umweltfaktoren, Ethnizität, Patientenalter) notwendig sind, um maligne Transformation durch LR‐HPV‐Typen wie HPV42 zu induzieren.

Mit dem aktuell verfügbaren nonavalenten HPV‐L1‐Impfstoff können mehr als 90% aller HPV‐induzierten Tumoren und anogenitaler Warzen verhindert werden, nicht jedoch HPV42‐induzierte Läsionen. Auch nach der erfolgreichen Elimination des Zervixkarzinoms und anderer HPV‐bedingter Läsionen in Ländern mit hohen HPV‐Impfraten muss weiterhin an HPV42 als Auslöser von AGW und seltenen digitalen und zervikalen Dysplasien und Karzinomen gedacht werden, da HPV42 von den momentan verfügbaren HPV‐Impfstoffen nicht abgedeckt wird. Zukünftig lassen sich HPV42‐induzierte Tumoren möglicherweise durch sich in Entwicklung befindende Pan‐HPV‐L2‐Impfstoffe verhindern.^26^, ^27^

## DANKSAGUNG

Open access Veröffentlichung ermöglicht und organisiert durch Projekt DEAL.

## INTERESSENKONFLIKT

Keiner.
